# The impact of EGFR mutations on the incidence and survival of stages I to III NSCLC patients with subsequent brain metastasis

**DOI:** 10.1371/journal.pone.0192161

**Published:** 2018-02-15

**Authors:** Wei-Yuan Chang, Yi-Lin Wu, Po-Lan Su, Szu-Chun Yang, Chien-Chung Lin, Wu-Chou Su

**Affiliations:** 1 Department of Internal Medicine, National Cheng Kung University Hospital, College of Medicine, National Cheng Kung University, Tainan, Taiwan; 2 Institute of Clinical Medicine, National Cheng Kung University Hospital, College of Medicine, National Cheng Kung University, Tainan, Taiwan; 3 Department of Nursing, National Cheng Kung University Hospital, College of Medicine, National Cheng Kung University, Tainan, Taiwan; Seoul National University College of Pharmacy, REPUBLIC OF KOREA

## Abstract

Previous studies have demonstrated the association between EGFR mutations and distant metastasis. However, the association for subsequent brain metastasis (BM) in stages I-III non-small cell lung cancer (NSCLC) patients remains inconclusive. We conducted a retrospective analysis to clarify the impact of EGFR mutations on the incidence of BM and associated survival in patients with stage I-III NSCLC. A total of 491 patients screened for EGFR mutations were retrospectively enrolled. Brain MRI or CT was used to detect the BM. Cumulative incidence of subsequent BM and overall survival (OS) after diagnosis of BM were estimated by the Kaplan-Meier method and compared using log-rank test. We performed Cox proportional hazard regression for predictors of subsequent BM and determinants of OS after BM. The cumulative incidence of BM seemed higher in patients harboring EGFR mutations than those without EGFR mutations although it did not reach statistical significance (hazard ratio [HR] = 1.75, 95% confidence interval [CI] = 0.73~1.81). After adjusting possible confounders, including age, smoking, stage, and tumor size, EGFR mutation became one of the predictors for subsequent BM (HR = 1.89, 95% CI = 1.12~3.17, *p* = 0.017). Though there was no statistical difference in survival after BM between patients with EGFR mutations and wild-type EGFR (median survival: 17.8 vs. 12.2 months, HR = 0.79, 95% CI = 0.45–1.40), patients with EGFR 19 deletion (Del) tended to have a longer survival after BM than the non-EGFR 19 Del group (median survival: 29.4 vs. 14.3 months, HR 0.58, 95% CI = 0.32–1.09, *p* = 0.089). In conclusion, our data suggested EGFR mutation to be one of the predictors for subsequent BM in stage I-III patients. Given the small sample size, more studies are warranted to corroborate our results.

## Introduction

Lung cancer is the leading cause of cancer-related deaths worldwide; in 2016, there were 158,080 lung cancer deaths in the USA alone [1]. In recent years, advances in our understanding of molecular abnormalities in lung cancer has helped define disease subgroups and develop specific molecular targets in the presence of driver mutations, thus providing valuable information for cancer treatment. The administration of epidermal growth factor receptor tyrosine kinase inhibitors (EGFR- TKIs), such as gefitinib, erlotinib and afatinab, is a major breakthrough in the management of advanced non-small cell lung cancer (NSCLC) [2]. EGFR mutation has been demonstrated to be the strongest predictor for the benefits of these EGFR-TKIs [3], which have shown to be superior to chemotherapy in terms of overall response rate (ORR), progression-free survival (PFS), and quality of life in untreated patients with EGFR mutation-positive NSCLC [2, 4–11]. Despite advances in systemic therapy and improvements in survival for advanced NSCLC, brain metastasis (BM) remains an important cause of morbidity and mortality. Nearly 50% of patients with metastatic NSCLC will develop BM during their disease courses [12]. In addition, the prognosis for patients with BM remains poor. The median overall survival (OS) was around 2–3 months among patients treated with systemic corticosteroids alone, and 3–6 months for those with whole brain radiation therapy (WBRT) [13, 14]. Though some studies suggested that patients with EGFR mutations had a higher incidence of BM compared with those with wild-type EGFR [15–17], others showed no significant association [18–21]. The definite association for BM in early-stage NSCLC patients is not fully understood due to the small sample size and lower proportion of patients available for EGFR mutation analyses in these studies. On the other hand, multiple case reports have described favorable outcomes with new or recurrent BM to EGFR TKI therapy, particularly in patients with sensitizing EGFR mutations [22–26]. Although the development of brain metastases in general predicts a poor outcome in lung cancer, it is not known whether EGFR mutation-positive patients with brain metastases have a better prognosis as compared to EGFR mutation-negative patients, especially those in stages I to III lung cancer.

The purpose of this study was to examine the significance of EGFR mutations on the incidence of brain metastases in a population of patients with a stage I to III lung cancer. We also evaluate the survival after the diagnosis of BM in relation to EGFR mutation status.

## Materials and methods

### Patients

The study was reviewed and approved by the Review Board and Ethics Committee of National Cheng Kung University Hospital (A-ER-105-327, S1 Fig) and all data were fully anonymized and the requirement for written informed consent was waived, given this study’s retrospective nature. This research was carried out in accordance with approved guidelines and the Declaration of Helsinki. We retrospectively reviewed patients between January 2010 and June 2016. The inclusion criteria for the study population consisted of patients with pathologically confirmed non-small cell lung cancer and receiving treatment at National Cheng Kung University Hospital. All patients received staging work-up including chest computed tomography (CT) scan and bone scan or brain images (CT or MRI) according to the clinical guidelines proposed by the National Comprehensive Cancer Network. The clinical stage was classified according to the tumor, node, metastasis (TMN) system proposed by the American Joint Committee on Cancer (7^th^ edition). Patients who were diagnosed as having stage IV disease during initial staging work-up were excluded.

### Data collection and follow-up

The inpatient and outpatient medical records of all patients were reviewed, and we collected data regarding the demographic and clinical characteristics, which include patient gender, age, smoking history, clinical/pathological stage, size of primary lung lesion, pathological subtype, treatment modalities, use of targeted therapy, date of initial diagnosis, date of subsequent BM, BM treatment, EGFR mutations, and time to recurrence, death date, and cause of death. Each patient was followed up until March 1, 2017. The presence of BM was defined as the presence of one or more enhanced lesions on CT or brain magnetic resonance imaging (MRI) and diagnosed when patients became symptomatic. Patients with lepto-meningeal metastases were also identified as BM. The time to subsequent BM was defined as the time between the date of initial diagnosis and the date of BM diagnosis; whereas the survival after diagnosis of BM was followed from the date of BM diagnosis to the date of death or being censored.

### EGFR mutations analysis

Tumor tissue from primary lung tumors were obtained for *EGFR* mutation analysis. Tissue samples that consisted of >80% tumor content, as determined via microscopy with hematoxylin and eosin staining, were selected for the study. DNA was extracted using the QIAcube automated extractor (Qiagen) with the QIAamp DNA FFPE tissue kit (Qiagen) and eluted in ATE (QIAmp Tissue Elution) buffer (Qiagen), according to the manufacturer’s instructions. Macrodissection was performed to enrich the final proportion of tumor DNA for analysis. The presence of EGFR mutations was determined using the EGFR PCR Kit (EGFR RUO Kit) and therascreen EGFR RGQ PCR Kit (EGFR IVD Kit). These kits combine Scorpions and ARMS technologies to detect the mutations using real-time quantitative PCR. Approximately 25 ng of DNA was loaded to each well and the assay was done according to the manufacturer’s instructions [27]. This assay system was designed to detect the common and uncommon EGFR mutations, including 19 deletions in exon 19, 3 insertions in exon 20, and the point mutations G719X (in exon 18), S768I (in exon 20), and L858R and L861Q (in exon 21). We then switched to the EGFR IVD Kit, which adds T790M (exon 20), an important TKI-resistant mutation. Analysis was done using the Rotor-Gene Q series built-in software version 2.0.3 (Build 2) for the EGFR RUO Kit and EGFR IVD Kit (Qiagen, Manchester, UK). Real-time curves were generated using FAM-labeled probes for both the control tube (exon 2, as a control) and each mutation in separate tubes. To calculate a ΔCT value for each mutation reaction, the following equation was used: [Mutation CT]–[Control CT] = ΔCT. Manufacturer-supplied ΔCT thresholds were used as LODs to call a mutation (≤ΔCT threshold is positive for mutation) [28].

### Statistical analysis

The frequencies and descriptive statistics of demographic and clinical variables were collected. Categorical variables were compared using a Chi-square test or Fisher exact test; whereas continuous variables were compared using Student’s *t*-test or Wilcoxon rank-sum test. The cumulative incidence of BM [29] and overall survival (OS) of patients after diagnosis of BM were estimated by the Kaplan-Meier method and compared using a log-rank test. We performed Cox proportional hazard regression models for predictors of subsequent BM and determinants of OS after BM diagnosis. The determination of predictors and prognostic factors is based on prior studies investigating the risk factors of brain metastasis or the prognostic factors of survival in early-stage lung cancer [30, 31]. Age at diagnosis, sex, smoking status, tumor stage, tumor size, and EGFR mutations, were chosen as the predictors and prognostic factors. Statistical Analysis System® software version 9.4 (SAS Institute, Cary, North Carolina, USA) was used to perform the analysis. All the reported *p*-values are two-sided.

## Results

### Patient characteristics

A total of 491 patients were enrolled in this study. The demographic and clinical characteristics are summarized in Table 1. Among these patients, 280 (57%) had EGFR mutations and 211 (43%) had wild-type EGFR. Among patients with EGFR mutations, 97 (34.6%) had exon 19 deletions, 152 (54.3%) had L858R substitution, and 31 (11.1%) had mutations in other sites or double mutations. EGFR mutations were predominantly found in adenocarcinoma (270 patients, 96.4%). There were higher proportions of patients with EGFR mutations who were female (59.3% vs. 35.5%, *p* = 0.019), non-smokers (77.1% vs. 48.8%, *p* < 0.001), and older than 60 years, (61.1% vs. 38.9%, *p* = 0.031). In addition, a higher proportion of patients with EGFR mutations had a tumor size of less than 30mm (60.4% vs. 43.6%, *p* < 0.001) and earlier stages (*p* < 0.001).

**Table 1:** Basic characteristics.

Variables	Total (%) N = 491	EGFR Mutation Status, n (%)		*p*
		WT n = 211	Mutant n = 280	
Gender				0.019
Female	241 (49.1)	75 (35.5)	166 (59.3)	
Male	250 (50.9)	136 (64.5)	114 (40.7)	
Age				0.031
≥60	293 (59.7)	122 (57.8)	171 (61.1)	
<60	198 (40.3)	89 (42.2)	109 (38.9)	
Mean	62.8	61.9	63.4	
Smoking History				< 0.001
No	319 (65.0)	103 (48.8)	216 (77.1)	
Yes	172 (35.0)	108 (51.2)	64 (22.9)	
Stage				< 0.001
IA	144 (29.3)	56 (26.5)	88 (31.4)	
IB	86 (17.5)	26 (12.3)	60 (21.4)	
IIA	22 (4.5)	7 (3.3)	15 (5.4)	
IIB	22 (4.5)	8 (3.8)	14 (5.0)	
IIIA	113 (23.0)	45 (21.3)	68 (24.3)	
IIIB	104 (21.2)	69 (32.7)	35 (12.5)	
Pathology				
Adeno	444 (90.4)	174 (82.5)	270 (96.4)	
SqCC	19 (3.9)	18 (8.5)	1 (0.4)	
Others	28 (5.7)	19 (9.0)	9 (3.2)	
Tumor size				< 0.001
≤30mm	261 (53.2)	92 (43.6)	169 (60.4)	
>30mm	225 (45.8)	115 (54.5)	109 (38.9)	
Mean	33.8	38.5	30.2	
ECOG				0.482
0	373 (76.0)	153 (72.5)	220 (78.6)	
1	98 (20.0)	49 (23.2)	49 (17.5)	
2	15 (3.1)	7 (3.3)	8 (2.9)	
>2	5 (1.0)	2 (0.9)	3 (1.1)	

### Risk factors for BM

**Fig 1:**
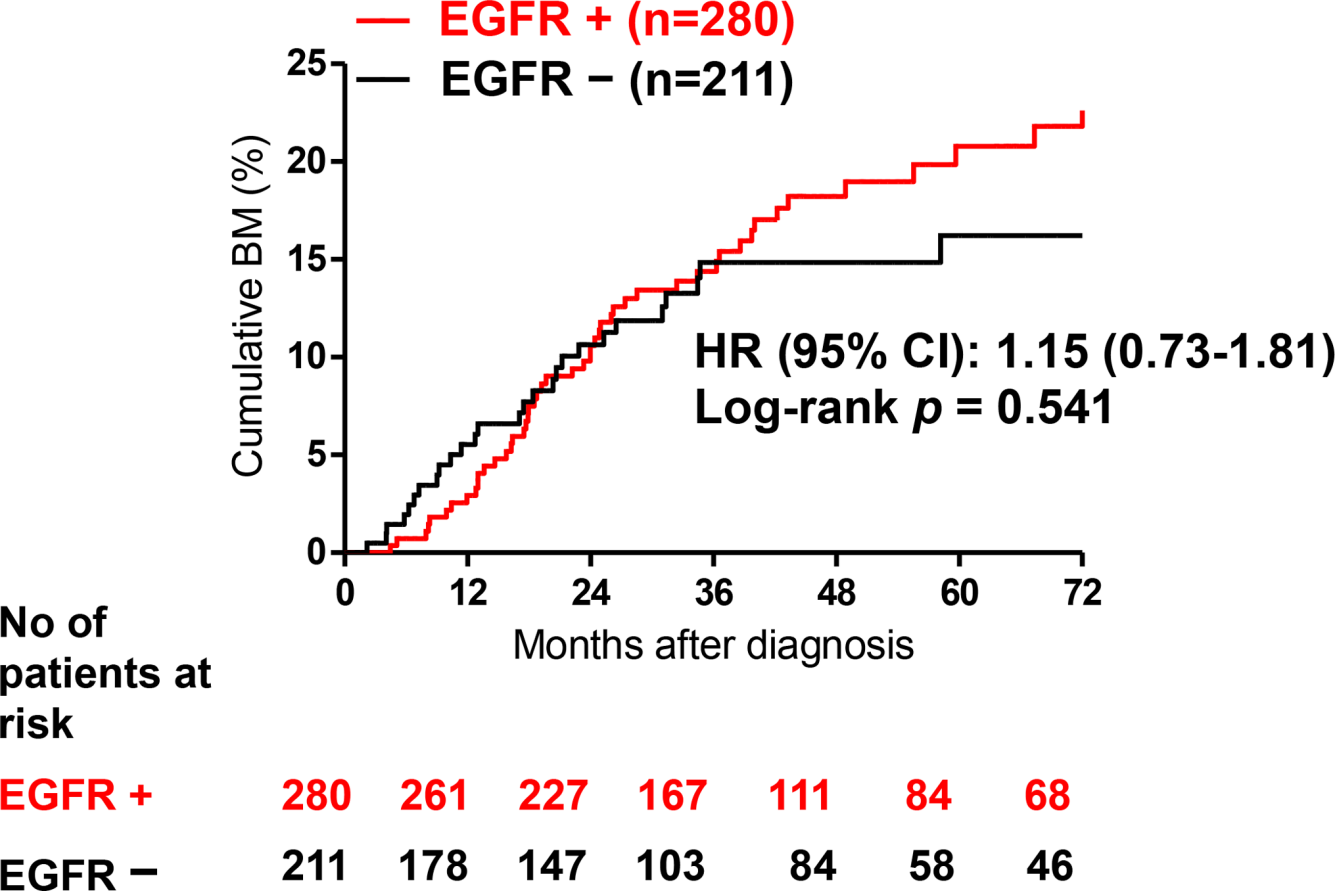
Cumulative incidence of brain metastasis (BM) in EGFR mutant versus wild-type patients.

The cumulative incidence of BM seemed higher in patients harboring EGFR mutations than those without EGFR mutations (Fig 1); however, it did not reach statistical significance. Cox proportional hazards models were conducted to adjust possible confounders of subsequent BM (Table 2). After adjusting possible confounders, including age, smoking, stage, and tumor size, EGFR mutation was one of the predictors for subsequent BM (HR = 1.89, 95% CI = 1.12~3.17, *p* = 0.017).

**Table 2:** Cox proportional hazard regression models for predictors of subsequent BM.

Variables	Total N = 491	Univariate	Multivariate		
		*p*	*p*	HR	95%CI
Sex		0.493	0.644	0.872	0.488–1.559
Female	241				
Male	250				
Dx age (each one year older)		0.011	0.006	0.968	0.947–0.991
Smoking		0.014	0.016	2.062	1.147–3.707
No	319				
Yes	172				
Stage					
IA	144				
IB	86	0.370	0.710	1.207	0.449–3.246
IIA	22	0.078	0.147	2.383	0.737–7.710
IIB	22	0.025	0.065	3.194	0.932–10.946
IIIA	113	<0.001	<0.001	4.078	1.925–8.636
IIIB	104	<0.001	<0.001	4.854	2.113–11.154
Tumor size		0.002	0.171	1.451	0.851–2.475
≤30mm					
>30mm					
EGFR mutation		0.511	0.017	1.885	1.120–3.171
Wild-type	211				
Mutant	280				

### Overall survival after BM and associated factors

**Fig 2:**
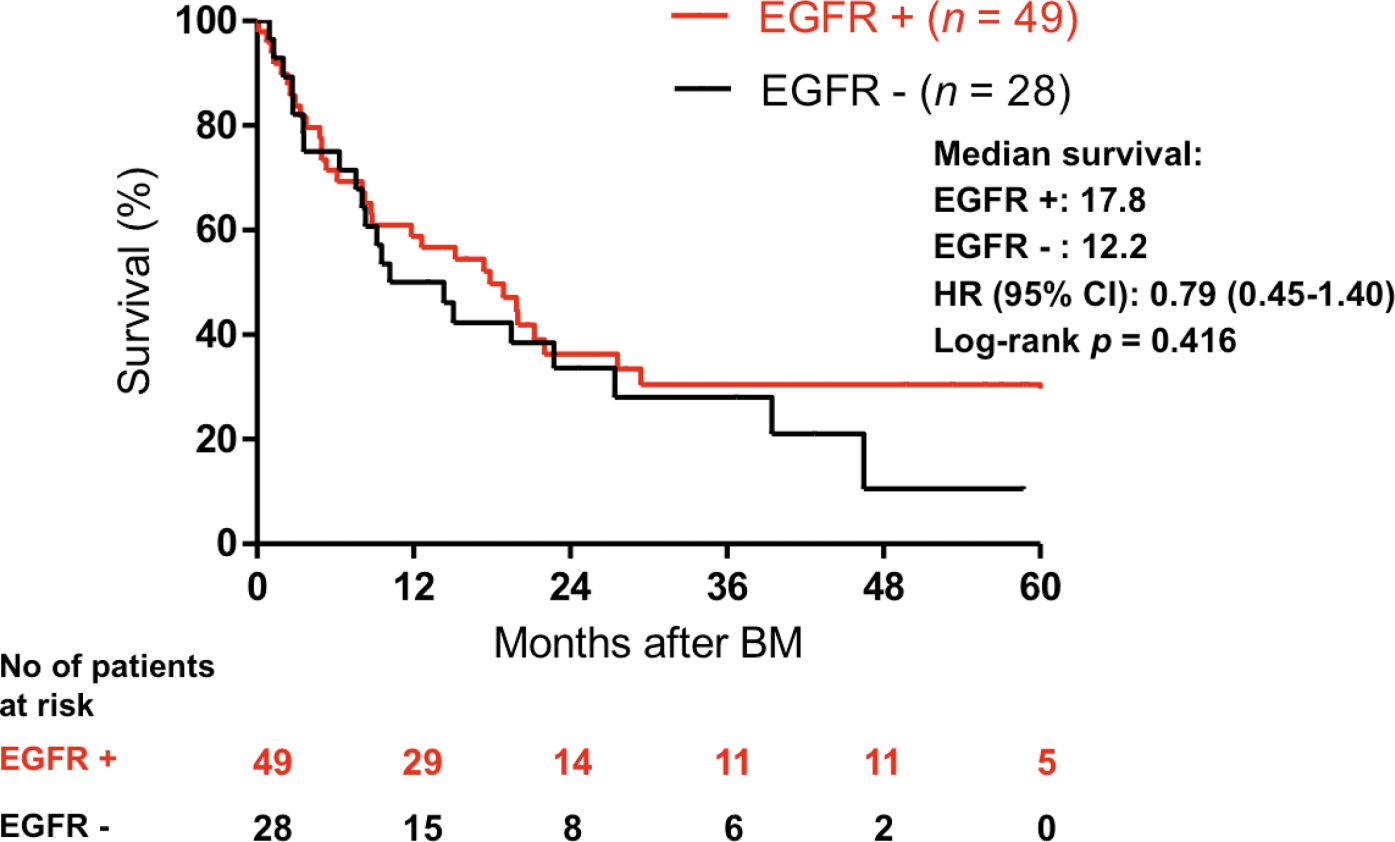
Kaplan-Meier curve for overall survival in patients with mutant EGFR mutation versus those with wild type EGFR after the diagnosis of brain metastasis.

**Fig 3:**
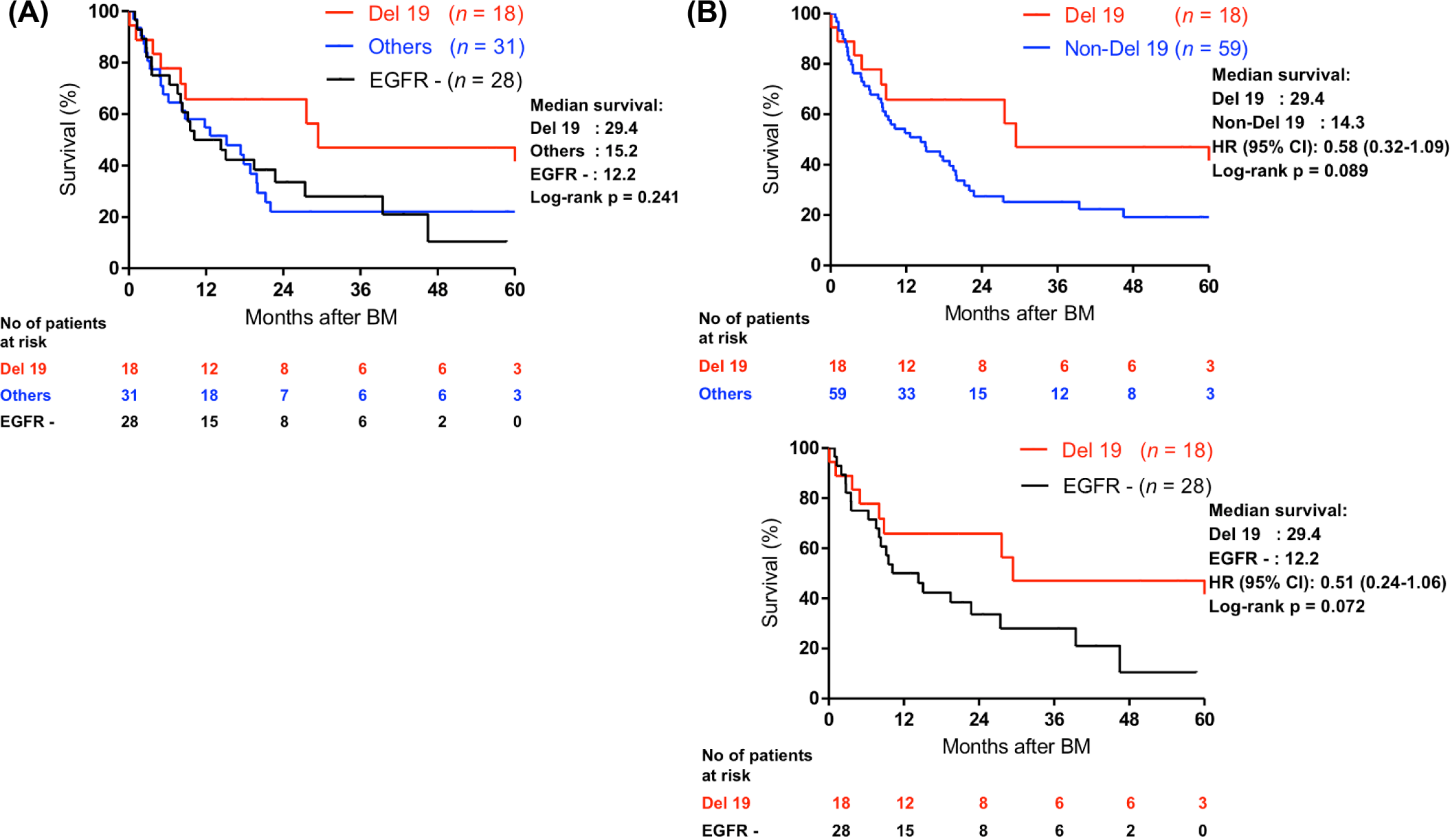
Kaplan-Meier estimations for overall survival in patients with different EGFR mutation status after the diagnosis of brain metastasis. (A) Exon 19 deletions versus other mutations and wild-type. (B) Exon 19 versus other mutations and exon 19 deletions versus wild type EGFR.

Though patients with EGFR mutations tended to have a longer OS after BM than patients with wild-type EGFR (Fig 2), it did not reach statistical significance (median survival: 17.8 vs. 12.2 months, HR = 0.79, 95% CI = 0.45–1.40). The age when patients were diagnosed with BM was the only significant prognostic factor of survival in the univariate analysis (Table 3). Sex, smoking history, stage, tumor size, EGFR mutations and whole brain radiotherapy had no statistical influence on survival. Previous studies revealed that patients with exon 19 deletions were associated with a longer progression-free survival compared to those with other mutations [32]. We therefore investigated if patients with EGFR 19 deletions had a longer OS after BM diagnosis in comparison with other mutations or wild type EGFR. Patients with exon 19 deletions had a longer median survival than that for patients harboring other EGFR mutations (29.4 months versus 14.3 months, HR = 0.58 (95% CI: 0.32–1.09)) and wild-type EGFR (29.4 months versus 12.2 months, HR = 0.51 (95% CI: 0.24–1.06)), but the differences were not statistical significant (Fig 3).

**Table 3:** Cox proportional hazard regression models for prognostic factors of survival after diagnosis of BM.

Variables	Total N = 78	Univariate	Multivariate		
		*p*	*p*	HR	95%CI
Sex		0.247	0.567	0.796	0.364–1.740
Female	37				
Male	41				
Dx age (each one year older)		0.032	0.010	1.036	1.009–1.064
Smoking		0.088	0.190	1.573	0.800–3.091
No	42				
Yes	36				
Stage					
IA	11				
IB	8	0.926	0.792	0.852	0.260–2.794
IIA	4	0.288	0.209	2.605	0.584–11.616
IIB	4	0.633	0.471	1.910	0.329–11.095
IIIA	28	0.568	0.554	0.757	0.301–1.905
IIIB	23	0.114	0.128	2.050	0.813–5.170
Tumor size		0.367	0.609	0.827	0.398–1.716
≤30mm	35				
>30mm	43				
EGFR mutation status 1		0.417	0.304	0.687	0.335–1.407
Wild-type	29				
Mutant	49				
EGFR mutation status 2		0.095	0.192	0.598	0.276–1.294
Wild-type	29				
Exon 19 deletion	18	0.118	0.139	0.486	0.187–1.265
Other mutations	31	0.938	0.471	0.765	0.368–1.587
Radiation therapy		0.157	0.422	0.513	0.100–2.619
No	22				
Yes	56				

## Discussion

In this study, we retrospectively reviewed and evaluated the different characteristics of BM according to the EGFR mutation status in patients with NSCLC. Although it did not reach statistical significance, we found patients with EGFR mutations seemed to have a higher cumulative incidence of BM than patients with wild-type EGFR (Fig 1). And EGFR mutation, a younger age, history of smoking, and locally advanced diseases predicted subsequent BM using Cox proportional hazard regression (Table 2). The median survival after diagnosis of BM tended to be longer in patients with EGFR mutations than those with wild-type EGFR and patients with exon 19 deletions had a median survival twice longer than that of patients who harbored other EGFR mutations or wild-type EGFR. However, the differences were also not statistical significant.

As EGFR mutant lung cancer patients survive longer because of the use of EGFR-TKIs, it would be unclear whether EGFR mutant lung cancer patients have BM due to their longer observation period or because EGFR mutant cancer cells tend to invade the brain. However, only stages I to III non-small cell lung cancer (NSCLC) patients were enrolled in our study, and most of these patients did not receive EGFR-TKIs. The effect of treatment with EGFR-TKIs on the incidence of BM would thus be minimal. The literature on the relationship between EGFR mutation status and subsequent brain metastases of stages I to III remains limited and inconclusive (Table 4) [21, 33–35]. Akamatsu *et al*. investigated the impact of outcomes according to EGFR mutation status in patients with stage III Adenocarcinoma, and found that those with EGFR mutations tended to develop BM as compared to those with wild-type EGFR after concurrent chemoradiotherapy (6/13 versus 4/31, *p* = 0.04). However, whether the EGFR mutation status was the independent factor could not be clarified due to the small sample size (*n* = 10) [33]. Stanic *et al*. investigated the correlation between EGFR mutation status and subsequent BM, and showed that EGFR status had no influence upon the cumulative incidence of this. Tanaka *et al*. investigated the impact of EGFR mutations on the efficacy of concurrent chemoradiation therapy (CRT), and found that concurrent CRT resulted in a shorter progression-free survival in EGFR-mutant stage III adenocarcinoma patients than in wild-type patients, mainly because of the distant metastasis. However, the correlation between EGFR mutation status and subsequent BM metastasis was not clarified. Yagishita *et al*. found that EGFR mutation is associated with a longer local control after definitive chemoradiotherapy in patients with stage III nonsquamous non-small-cell lung cancer. Though more patients with EGFR mutations developed brain relapses than those with wild-type EGFR (16 versus 12), the correlation was not further investigated [35]. A summary of the studies directly examining EGFR mutation status and brain metastases is presented in Table 4. We have the largest number of patients in comparison with other works, since EGFR mutation status was checked during the study period. The mean age in our study does not differ from that of other studies, although there was a higher proportion of female patients in our group as compared to other works. However, this ratio is acceptable, since most cancer types were adenocarcinomas, and this is compatible with the findings of another study investigating the association between adenocarcinoma and EGFR mutation in Taiwan [36]. We also noted those patients with EGFR mutation tended to be older and their brain tumor size tended to be smaller. These are important points, since such factors will affect BM and survival. According to the analysis of Taiwan’s nationwide lung cancer registry focusing on epidermal growth factor receptor mutation and smoking status, the EGFR mutation rate of younger lung cancer patients was significantly lower than that in the older group [37]. Moreover, in a study with a total of 401 Chinese NSCLC patients (280 males and 121 females) investigating the correlation between EGFR mutations and incidence of distant metastases and tumor size in patients with non-small-cell lung cancer, the tumor size in EGFR mutation group was significantly smaller than that in the wild-type group (*p*< 0.001), as shown in our study [38]. The EGFR mutation rate (57%) found in the current work is higher than in the other four studies. However, according to a recent systematic review and global map of EGFR mutation incidence in NSCLC [39], the frequency of EGFR mutations among adenocarcinoma patients in the Asia-Pacific area ranges from 20% to 76%, and the mean frequency is 57% in Taiwan. Our study further identified that EGFR mutation was independently associated with subsequent BM (odds ratio 2.246) in a multiple logistic regression model. Other risk factors, such as younger age and locally advanced diseases, have been demonstrated to be associated with BM in other studies [40, 41]. As for the correlation between smoking history and brain metastasis, we are the first work demonstrating their correlation. Recent studies have shown that smoking tobacco is associated with cancer metastasis [42, 43], but the associated mechanism underlying the correlation between metastasis remains unclear.

**Table 4:** Summary of studies examining the association between EGFR mutations and brain metastasis in patients with stages I to III NSCLC.

Author	Patient (*n*)	Country	Mean age	Sex (F, %)	EGFR mutation (%)	Stage (%)	Association between BM and EGFR mutations
Hiroaki Akamatsu [33]	44	Japan	65.2	27.3	29.5	III	Significant
Karmen Stanic [21]	245	Slovenia	N/A	N/A	30.6	I to III	Non-significant
Kosuke Tanaka [34]	104	Japan	62.0	38.0	28.0	III	Not mention
Shigehiro Yagishita [35]	198	Japan	60.0	30.2	17.0	III	Not mention
Current study	491	Taiwan	62.8	49.0	57	I to III	Significant and independent

N/A: Not available

The molecular mechanism for the linkage between EGFR mutations and BM remains unclarified. It is proposed that EGFR downstream signaling and other pathways which activate EGFR signaling contribute the metastasis to the brain in patients harboring EGFR mutations. Mutant EGFR could induce IL-6 activation and then up-regulate the downstream gp130/JAK/STAT3 pathway [44], and STAT3 cooperates with microRNA-21 (miR-21) contributing to lung-to-brain metastases [45]. Moreover VEGF, which creates a favorable environment that promotes metastasizing to the brain, was found to be upregulated by STAT3 and EGFR [46]. Other pathways, such as Met [47], C/EBPβ-LIP/CUG-binding protein 1 (CUGBP1) [48] and phosphoinositide 3-kinase/protein kinase B / phospholipase C γ [49], have been shown to promote BM via activation by EGFR. However, more studies are needed to elucidate the exact role of EGFR mutation in BM at the molecular level.

The median survival from the diagnosis of BM to death was 15.2 months for all patients with BM. The EGFR mutation status seemed to influence the median survival time after BM (17.8 vs. 12.2 months) but with no statistical significance (HR 0.79, 95% CI = 0.45–1.40). Stanic [21] and Baek et al. [50] also investigated the impact of EGFR mutation median survival on patients with BM, and found that during the later course of the disease there was no significant difference between EGFR mutant and wild-type patients (p = 0.7 and *p* = 0.23, Table 5). Han et al. demonstrated that EGFR mutation is an independent predictive and prognostic risk factor for BM, and a positive predictive factor for OS in patients with BM [51]. However, whether EGFR mutation served as an independent prognosis factor was not revealed. In another study with more patients enrolled, the EGFR mutation status strongly influenced the median survival time if BM had been already discovered at diagnosis [52, 53]. However, whether the relationship can be observed in larger cohorts of patients with stages I to III remains unclear. We also found EGFR mutations were more common among elderly patients, and that such patients tended to have worse survival after diagnosis of BM compared to younger patients, with borderline significance (HR = 1.036). However, the benefits of TKIs in NSCLC with regard to elderly and younger patients, both in terms of PFS and OS, remain controversial [54, 55].

**Table 5:** The four studies selected for examining the association between EGFR mutations and overall survival of NSCLC patients with subsequent brain metastasis.

Author	Patient EGFR^M^	Patient EGFR^W^	Medium OS EGFR^M^	Medium OS EGFR^W^	Hazard ratio	Exon 19 vs. Other mutation and wild type (HR)
Karmen Stanic [21]*	26	64	N/A	N/A	N/A (p = 0.7)	N/A
Guang Han [51]*	48	28	23.8	14.2	N/A (p = 0.028)	N/A
Min Young Baek [50]	7	13	14.5	2.5	N/A (p = 0.23)	N/A
Current study	49	28	17.8	12.2	0.687 (p = 0.30)	0.58 (*p* = 0.089)

*EGFR mutation was an independent prognosis factor under univariate and multivariate analysis.

A previous study has showed that exon 19 deletions are associated with prolonged survival among EGFR-mutant metastatic lung adenocarcinoma patients treated with EGFR-TKI [32]. Theoretically, there may be more BM observed throughout the disease course of those patients with exon 19 deletions. However, our analysis did not find any difference in subsequent BM in stages I to III NSCLC between exon 19 deletions and other mutations (19% vs. 20%). On the other hand, the prognostic value of different EGFR mutations in resected NSCLC remains controversial. In our study, patients with exon 19 deletions tended to have a longer survival after BM (29.4 months) than patients with other mutations (15.2 months) or wild-type EGFR (12.2 months). The difference was not significant after adjusting for other factors. Larger retrospective studies are needed to verify if stages I to III patients with exon 19 deletions and subsequent BM has better survival with investigation of associated mechanism.

The efficiency of systemic chemotherapy combined whole brain radiotherapy (WBRT) for the treatment of patient with BM is limited, with reported response rates ranging from 40–60% (overall survival [OS] 6–12 months) [56, 57]. Conversely, response rates of brain metastases to EGFR tyrosine kinase inhibitor (TKI) treatment in patients with NSCLC harboring EGFR mutations reach 60–80%, with median OS around 15–20 months, demonstrating an improved clinical outcome [58]. The different response rates to BM come from the good efficiency of EGFR-TKI in passing through the blood brain barrier and targeting the BM of NSCLC patients harboring sensitive EGFR mutations [59, 60]. Besides, some patients receiving WBRT developed cognitive problems, particularly in terms of short-term memory, which were not observed in patients receiving EGFR-TKI [61]. On the other hand, though recent research demonstrated that advanced NSCLC patients with exon 19 deletion might have longer PFS compared to those with L858 mutation after first-line EGFR-TKIs [32, 62], the reason for the observed difference remained inconclusive. Some mechanisms suggested by preclinical studies were proposed to explain the difference in efficacy of EGFR TKIs according to EGFR mutation subtype. Carey et. al. performed an in vitro kinetic analysis of peptide phosphorylation reactions with purified intracellular domains from EGFR wild-type, L858R, and EGFR del746-750. The results of a kinetic assay indicated a higher affinity of gefitinib and erlotinib for recombinant EGFR with the exon 19 deletion than that with the L858R mutation [63]. Another study showed cell lines with different EGFR mutations expressed different EGFR phosphorylation status and downstream signaling before and after EGFR-TKI treatment. The human embryonic kidney cell (293) cell line was transfected with a vector with inserts containing the entire length of EGFR with L858R or EGFR del746–750, and the baseline levels of EGFR autophosphorylation were not different in both conditions. However. gefitinib induced a more marked decrease in EGFR autophosphorylation at tyrosine residues 1173, 845, and 1045 and a lesser decrease at Y992 in del746–750 cells, compared with the autophosphorylation levels in L858R cells. The phosphorylation levels of major downstream signals of EGFR, including Akt and Erk1/2, decreased more sharply in del746–750 cells than in L858R cells [64]. Therefore, the different phosphotyrosine patterns between these two mutations may be associated with differential response durations of the EGFR TKIs. A recent study further showed that the exon 19 deletion group had a longer median PFS than the L858R mutation group (6.7 vs. 3.9 months, p<0.001) in patient with BM [65]. Some research showed that NSCLC patients with exon 19 deletion had more and smaller metastases with a reduced extent of peritumoral brain edema compared with patients with wild-type EGFR alleles. The characteristics of BM in patients with L858R mutation were also similar to those of the metastases in wild-type patients [66]. Recent clinical study showed the survival of patients with Exon 19 Del is better than those with L858R because the former group developed higher proportion of EGFR T790M which was correlated with a better prognosis than other acquired mutations such as met positive or KRAS/PIK3CA/ALK-altered population [67]. However, more efforts are needed to investigate if these molecular mechanisms and characteristics of BM are the key issues of the more favorable efficacy in terms of exon 19 deletion compared with L858R mutation in patients with BM.

There are several limitations to this study that should be noted. First, it is a retrospective study from a single-institution and not all patients received testing of EGFR mutation during the enrolled period (see S2 Fig and S1 Table). Though EGFR mutation may be studied on tumor resection or on tumor recurrence, most of the tumors were checked for EGFR mutation at initial diagnosis. Second, there are many significant differences with regard to clinical characteristics between the EGFR mutations and EGFR wild-type groups, including case number, age, stage distribution and tumor size. However, EGFR mutations remained one of the independent risk factors after multiple regression to adjust for confounders. Third, we did not investigate if the choice of first line TKIs affect the prognosis of patient with subsequent BM with Exon 19 Del. A previous study found overall survival was significantly longer for patients with Exon 19 Del-positive tumours treated with irreversible first-line TKI than in the chemotherapy group. And the survival difference was not observed in other reversible first-line TKIs [68]. Fourth, the incidence of BM diagnosis may be underestimated because serial brain image examination was not part of standard follow-up. It is thus possible that asymptomatic disease was not detected. Fifth, we did not evaluate the influence of other genetic changes, such as KRAS mutation, Met amplification, or EML4-ALK translocation. However, this potential bias may be small because the frequencies of other driver mutations are relatively low, and more than one driver mutation is rarely found concurrently in the same tumor. Finally, there is a relatively low number of BM patients in this cohort, especially those with subsequent BM after 36 months of diagnosis (Fig 1), when compared to other studies aimed at examining the correlation between EGFR mutation and BM in late-stage patients. Therefore, the statistical significance may be over-estimated and a larger cohort is thus required to verify the difference in risk of subsequent BM and associated survival between EGFR mutation-positive and wild-type EGFR patients.

Previous studies have showed that a brain MRI is not indicated due to the low incidence of asymptomatic BM in patients with operable NSCLC [69]. Our results implied the importance of brain imaging, especially for patients with EGFR mutations, even those with stages I to III. Moreover, recent studies have highlighted the role of EGFR-TKIs in the adjuvant treatment of NSCLC [70, 71]; consequently, our study may provide a clue in selecting the EGFR-TKIs with a high concentration in brain in order to prevent a higher incidence of BM in these patients.

## Conclusion

Our data suggested that EGFR mutation is one of the predictive factors for the development of BM. Though it did not reach statistical significance, NSCLC brain-metastatic patients with exon 19 deletions tended to have a longer survival than those with other EGFR mutations and wild-type EGFR. These observations help delineate subsets of patients who tend to develop BM and who might reach a longer BM survival. Further studies designed to investigate the molecular and genetic factors that impact survival should help further improve our understanding of the heterogeneous outcomes in these patients.

## Supporting information

S1 Fig The approval of research plan and protocol from Institutional Review Board (IRB) and institutional ethics committee. (TIF)

S2 Fig Flow chart describing patients enrolled in the study. (TIF)

S1 Table Clinical characteristics of patients with or without EGFR mutation test. (DOCX)
