# Supplementary figures and images for: The impact of EGFR mutations on the incidence and survival of stages I to III NSCLC patients with subsequent brain metastasis
Source: PLoS One. 2018 Feb 15;13(2):e0192161. doi: 10.1371/journal.pone.0192161 (PMC5813924; doi:10.1371/journal.pone.0192161)

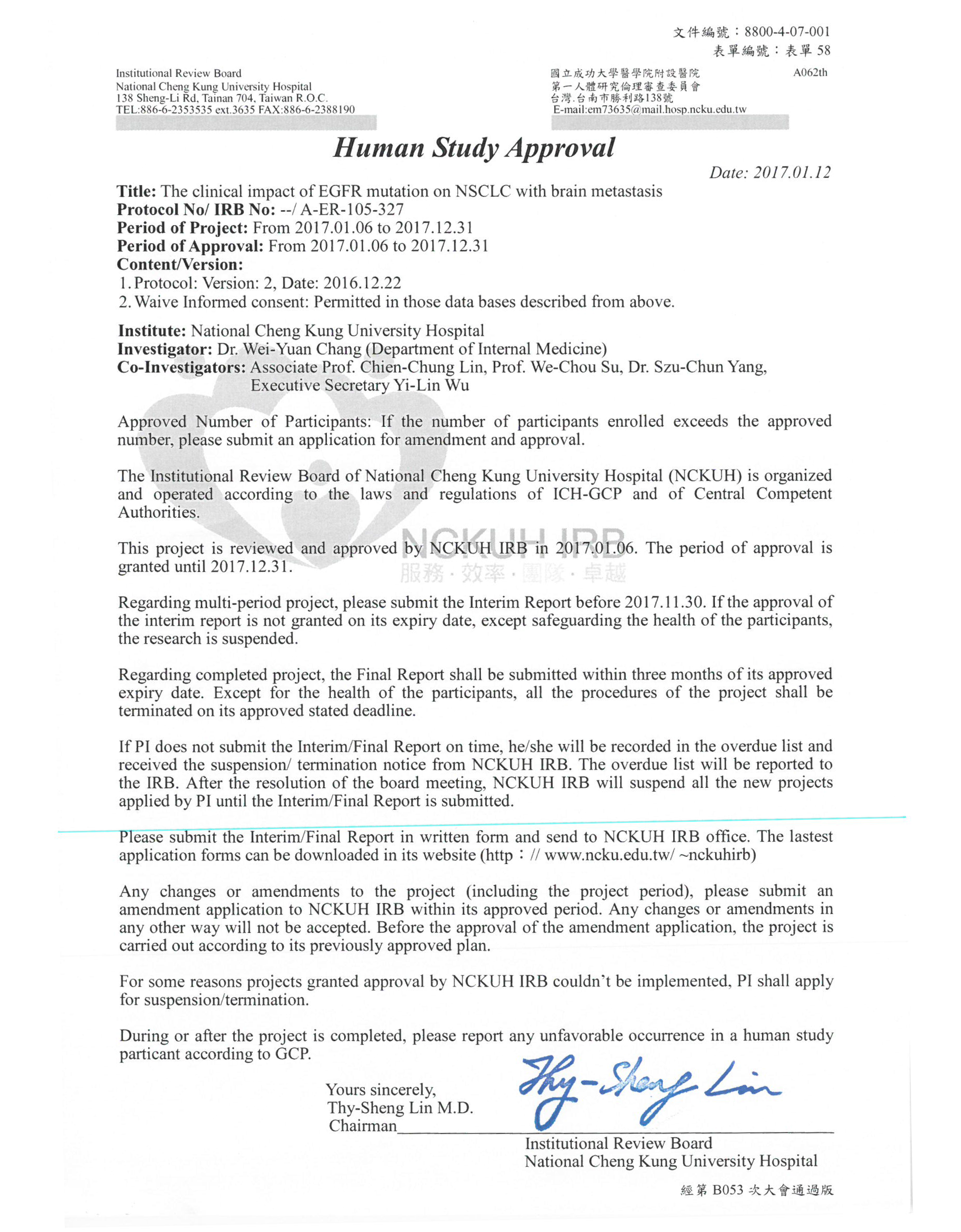

Supplement: S1 Fig — (TIF) [file pone.0192161.s001.tif]

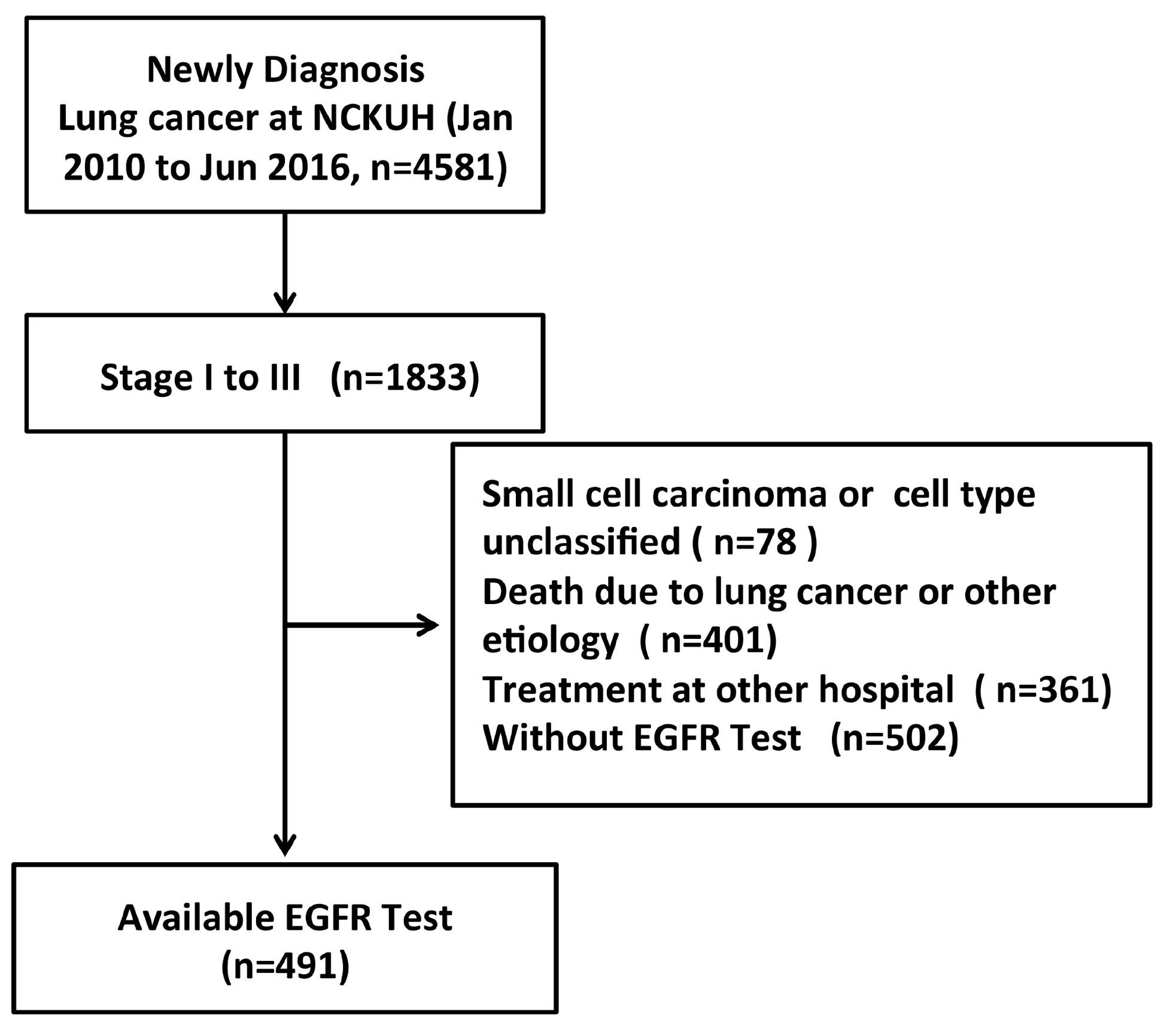

Supplement: S2 Fig — (TIF) [file pone.0192161.s002.tif]
