# Supplementary material for: The impact of EGFR mutations on the incidence and survival of stages I to III NSCLC patients with subsequent brain metastasis
Source: PLoS One. 2018 Feb 15;13(2):e0192161. doi: 10.1371/journal.pone.0192161 (PMC5813924; doi:10.1371/journal.pone.0192161)
Supplement: S1 Table — (DOCX) [file pone.0192161.s003.docx]

S1 Table Clinical characteristics of patients with or without EGFR mutation test.

|  | EGFR Test | | | |
| --- | --- | --- | --- | --- |
|  | Performed  (n=491) | | Not performed  (n=502) | |
| **Gender** |  |  |  |  |
| Female | 241 | 49.1% | 208 | 41.4% |
| Male | 250 | 50.9% | 294 | 58.6% |
| **Age** |  |  |  |  |
| ≥60 | 293 | 59.7% | 344 | 68.5% |
| <60 | 198 | 40.3% | 158 | 31.5% |
| mean | 62.8 |  | 65.4% |  |
| **Stage** |  |  |  |  |
| 1A | 144 | 29.3% | 182 | 36.3% |
| 1B | 86 | 17.5% | 76 | 15.1% |
| 2A | 22 | 4.5% | 15 | 3.0% |
| 2B | 22 | 4.5% | 24 | 4.8% |
| 3A | 113 | 23.0% | 83 | 16.5% |
| 3B | 104 | 21.2% | 122 | 24.3% |
| **Cell Type** |  |  |  |  |
| Adenocarcinoma | 444 | 90.4% | 256 | 51.0% |
| Squamous Cell Carcinoma | 19 | 3.9% | 71 | 14.1% |
| Others | 28 | 5.7% | 175 | 34.9% |
| **Smoking History** |  |  |  |  |
| Never-smoker | 319 | 65.0% | 258 | 51.4% |
| Ever-smoker | 172 | 35.0% | 239 | 47.6% |
| **Test Timing** |  |  |  |  |
| At initial diagnosis | 273 | 55.6% |  |  |
| At recurrence | 218 | 44.4% |  |  |
|  |  |  |  |  |
| **No Recurrence** | 188 | 38.3% | 286 | 57% |
| Adenocarcinoma | 177 |  | 177 |  |
| Squamous Cell Carcinoma | 3 |  | 67 |  |
| Others | 8 |  | 53 |  |
